# Development of GSH-Stimuli-Responsive Micelles Using a Targeted Paclitaxel Prodrug for Enhanced Anticancer Effect

**DOI:** 10.3390/pharmaceutics17040538

**Published:** 2025-04-21

**Authors:** Qian Ning, Guangping Yu, Wenkai Yi, Minhui Gu, Qianqian Xu, Zhiting Ye, Mengxia Zhang, Shengsong Tang

**Affiliations:** 1 College of Bioscience and Biotechnology, Hunan Agricultural University, Changsha 410127, China; ningqian@hnmu.edu.cn; 2 Hunan Province Key Laboratory for Antibody-Based Drug and Intelligent Delivery System, School of Pharmaceutical Sciences, Hunan University of Medicine, Huaihua 418000, China; 19896222892@163.com (G.Y.); 15773946992@163.com (W.Y.); 15367694226@163.com (M.G.); 15755656104@163.com (Q.X.); m17520395582@163.com (Z.Y.); 3 Institute of Pharmacy & Pharmacology, School of Pharmaceutical Science, University of South China, Hengyang 421001, China; 4 Department of Histology and Embryology, Hunan University of Chinese Medicine, Changsha 410128, China; 5 Department of Pharmacology, Ningxia Medical University, Yinchuan 750004, China

**Keywords:** micelles, co-delivery, synergistic effect, oligo-hyaluronic acid, microfluidic self-assembly

## Abstract

**Background:** Cancer ranks as a leading cause of death worldwide. It is urgent to develop intelligent co-delivery systems for cancer chemotherapy to achieve reduced side-effects and enhanced therapeutic efficacy. **Methods:** We chose oligo-hyaluronic acid (oHA, a low molecular weight of HA) as the carrier, and adriamycin (ADM) and paclitaxel (PTX) as the co-delivered drugs. The oHA-ss-PTX macromolecular prodrug was synthesized by introducing glutathione-stimuli-responsive disulfide bonds through chemical reactions. Then, we constructed ADM-loading micelles (ADM/oHA-ss-PTX) in one step by microfluidic preparation. The delivery efficacy was evaluated comprehensively in vitro and in vivo. The biocompatibility of ADM/oHA-ss-PTX was assessed by hemolysis activity analysis, BSA adsorption testing, and cell viability assay in endothelial cells. **Results:** The resulting ADM/oHA-ss-PTX micelles possessed a dynamic size (127 ± 1.4 nm, zeta potential −9.0 mV), a high drug loading content of approximately 21.2% (PTX) and 7.6% (ADM). Compared with free ADM+PTX, ADM/oHA-ss-PTX showed enhanced blood stability and more efficiently inhibited cancer cell proliferation. Moreover, due to the CD44-mediated endocytosis pathway, a greater number of ADM/oHA-ss-PTX micelles were absorbed by A549 cells than by oHA-saturated A549 cells. In vivo experiments also showed that ADM/oHA-ss-PTX micelles had excellent therapeutic effects and targeting ability. These results show that ADM/oHA-ss-PTX micelles were a promising platform for co-delivery sequential therapy in CD44-positive cancer. **Conclusions:** In conclusion, these results convincingly demonstrate that ADM/oHA-ss-PTX micelles hold great promise as a novel platform for co-delivering multiple drugs. Their enhanced properties not only validate the potential of this approach for sequential cancer therapy in CD44-positive cancers but also pave the way for future clinical translation and further optimization in cancer treatment.

## 1. Introduction

Cancer is ranked as a leading cause of death globally and represents a significant impediment to the increase in life expectancy [1]. Lung cancer and breast cancer are among the most prevalent malignancies [2]. Surgery, radiotherapy and chemotherapy are the primary treatment modalities in current cancer management [3,4]. Chemotherapy is still currently the most commonly used treatment in various clinical applications. However, its non-selective nature gives rise to adverse effects such as bone marrow suppression and gastrointestinal reactions. Approximately 30% of patients discontinue treatment due to toxicity [5,6]. Additionally, drug resistance emerges as a major obstacle in cancer chemotherapy. Consequently, there is an imperative to enhance the efficacy of chemotherapy by integrating precise typing and multidisciplinary strategies.

Paclitaxel (PTX) and adriamycin (ADM) are among the most prevalently deployed chemotherapeutic agents in the realm of cancer treatment [7]. Nevertheless, high toxicity and low bioavailability as well as drug resistance significantly limit its clinical application. PTX, for instance, exhibits poor water solubility and bioavailability, and is associated with severe allergic reactions and neurotoxicity [8,9]. Similarly, ADM has been linked to cardiotoxicity and non-specific distribution, limiting its long-term application [8,9,10,11]. In recent years, in an attempt to surmount these obstacles, researchers have harnessed the power of nanotechnology and devised targeted delivery systems. For instance, functionalized nanocarriers based on liposomes, polymeric nanoparticles and hyaluronic acid (HA) have been extensively utilized to enhance tumor targeting and concurrently alleviate the adverse effects associated with the drugs [12,13]. Conversely, the development of pH-sensitive nanocarriers and the implementation of targeted ligand modification systems for adriamycin have effectively mitigated cardiotoxicity and enhanced tumor-specific delivery [14,15]. These strategies have considerably enhanced therapeutic efficacy and patient tolerability by optimizing the pharmacokinetic and distribution properties of the drugs.

Oligo-hyaluronic acid (oHA), a derivative of the naturally occurring polysaccharide, has demonstrated considerable advantages in the field of drug delivery systems in recent years [16]. HA, a well-known biocompatible polysaccharide, is ubiquitously distributed within the extracellular matrix of the human body [17,18]. oHA, the low-molecular-weight variant of HA, is distinguished by its diminutive molecular size and enhanced tissue-penetrating ability. In contrast to its high-molecular-weight counterpart, oHA not only retains excellent biocompatibility and biodegradability, but also exhibits an elevated cell-targeting proficiency and drug-encapsulation efficiency. oHA’s unique molecular structure enables it to target tumor cells and inflammatory sites via receptor-mediated endocytosis (e.g., CD44 and RHAMM receptors), which improves drug delivery precision and therapeutic efficacy [19,20,21,22]. In addition, oHA demonstrates favorable water solubility. This inherent property renders it amenable to chemical conjugation with a diverse array of substances (e.g., small molecule drugs, proteins, and nucleic acids) through either covalent or non-covalent linkages [23,24].

**Scheme 1:**
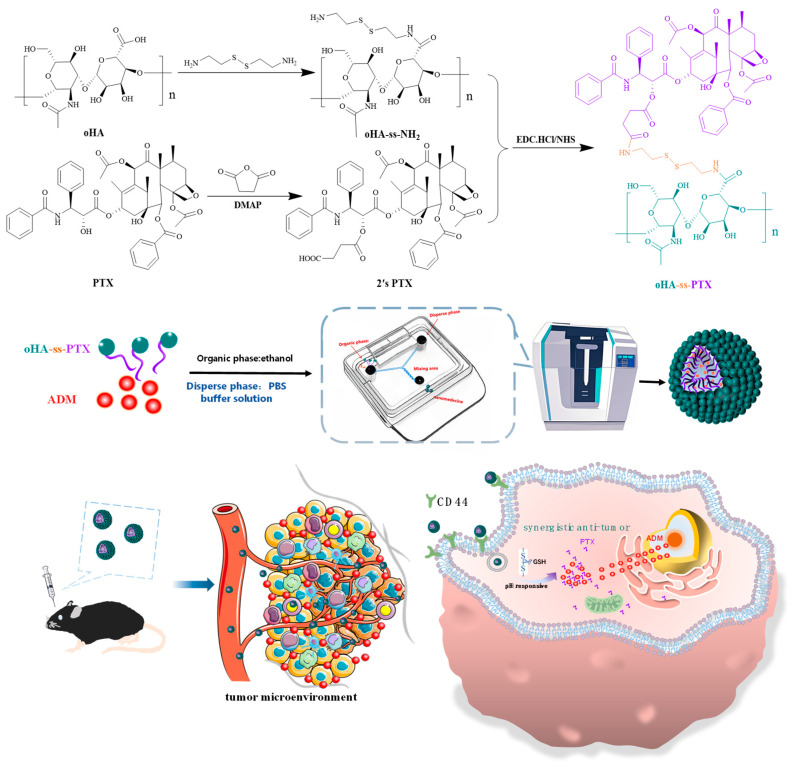
Schematic illustration of the ADM/oHA-ss-PTX for combination therapy.

In this work, we selected oHA as the carrier material and disulfide bond (-ss-) as the linker for the connection between PTX and oHA. The disulfide bond in cystamine functioned as a glutathione (GSH)-responsive switch to realize the stimuli-triggered drug release due to the massive GSH in tumor cell cytoplasm [25,26]. We synthesized amphiphilic prodrug oHA-ss-PTX, and prepared ADM/oHA-ss-PTX micelles loaded with PTX and ADM by microfluidic self-assembly. OHA has the ability to bind specifically to CD44, an activated cell surface glycoprotein, and could actively target tumor tissue and be internalized by cancer cells via CD44-mediated endocytosis [27,28]. Following internalization of the tumor cells, the system is stimulated by the tumor microenvironment, resulting in the orderly release of the chemotherapeutic drugs ADM and PTX. These drugs exert a synergistic antitumor effect (Scheme 1). The structure and nanofabrication process of the resulting ADM/oHA-ss-PTX were optimized, and comprehensive in vitro and in vivo evaluations were carried out.

## 2. Materials and Methods

### 2.1. Materials

PTX and ADM hydrochloride were procured from Wuhan Yuancheng Gongchuang Technology Co., Ltd. (Wuhan, China). OHA (Biomedical Grade, Mw = 3 kDa) was provided by Meilun Biotechnology Co., Ltd. (Dalian, China). Fetal bovine serum (FBS) and medium were acquired from Thermo Fisher Scientific Company(Waltham, MA, USA). CCK8 and DAPI were purchased from Sigma Company (Ridge, NJ, USA).

MCF-7 cells, A549 cells, and Human Umbilical Vein Endothelial cells (ECs cells) were provided by Stem Cell Bank, Chinese Academy of Sciences (Shanghai, China). Lewis lung carcinoma cell lines (LLC cells) were purchased from IMMOCELL (Xiamen, China).

Male C57BL/6 (6 week, 18–22 g) mice were purchased from Hunan SJA Laboratory Animal Co., Ltd. (Changsha, China). New Zealand white rabbits were supplied by the Animal Experimental Center (Hunan University of Medicine, Changsha, China). All animal experiments were carried out in strict accordance with the guidelines approved by the Ethics Committee of Hunan University of Medicine (2021A031731).

### 2.2. Synthesis of Amphiphilic Prodrug oHA-ss-PTX

#### 2.2.1. Synthesis of Redox-Sensitive oHA-ss-NH_2_

oHA-ss-NH_2_ was synthesized based on conjugation of cystamine to oHA by an amide bond. Briefly, cystamine dihydrochloride (1.46 mmoL) was dissolved in 15 mL mixed solution of methanol and triethylamine. After stirring the reaction at 37 °C, the solution was removed through a rotary evaporator, re-dissolved in double-distilled water (10 mL) and the solution was adjusted to pH 5.0. oHA (0.25 mmoL) were dissolved in double-distilled water, and then water solution of cystamine were slowly poured into it, simultaneously, add 1-ethyl-3-(3-dimethylaminopropyl) carbodiimide hydrochloride (EDC·HCl, 0.5 mmoL) and the solution was adjusted to pH 5.0. After water bath stirring the reaction at room temperature for 4 h, then the reaction solution was dialyzed in double-distilled water for 3 days and lyophilized dialysate for 2 days to give oHA-ss-NH_2_. The successful synthesis of oHA-ss-NH_2_ was subsequently confirmed by FT-IR spectra and NMR hydrogen spectroscopy. For oHA-ss-NH_2_, D_2_O was used as the solvent for NMR hydrogen spectroscopy. The synthetic route was illustrated in Scheme 1.

#### 2.2.2. Carboxylation of Paclitaxel (2′sPTX)

PTX was successfully carboxylated through an esterification reaction. PTX (0.1 mmoL), 4-dimethylaminopyridine (DMAP, 0.53 mmoL) and succinic anhydride (0.35 mmoL) were dissolved in the 25 mL dichloromethane and solution was stirred at 25 °C for 4 h. The reaction solution was washed three times with 0.5 mol/L diluted HCl and three times with saturated brine, and dried over anhydrous Mg_2_SO_4_. The final product was purified by silica gel column chromatography. The successful synthesis of 2′sPTX was subsequently confirmed by FT-IR spectra and NMR hydrogen spectroscopy. For 2′sPTX, CDCl_3_ was used as the solvent for NMR hydrogen spectroscopy. The synthetic route was illustrated in Scheme 1.

#### 2.2.3. Synthesis of Amphiphilic oHA-ss-PTX

The synthesis of oHA-ss-PTX was performed through amidation in which the carboxyl group of 2′sPTX was grafted to the oHA-ss-NH_2_. First, the carboxyl group of 2′sPTX was activated using catalyst. 2′sPTX (13.0 mg) was dissolved in the N, N-dimethylformamide to which EDC·HCl and NHS were added. Secondly, oHA-ss-NH_2_ (20 mg) was dissolved in formamide. Afterwards, the activated 2′sPTX solution was slowly dripped into formamide solution of oHA-ss-NH_2_. Finally, the reaction solution was placed in a 2 kDa dialysis membrane, dialyzed for one day in ethanol/water, and then dialyzed in double distilled water for 2 days. Lyophilizing the dialysate for 48 h yielded oHA-ss-PTX. The successful synthesis of oHA-ss-PTX was subsequently confirmed by FT-IR spectra and NMR hydrogen spectroscopy. For oHA-ss-PTX, a mixed solvent of D_2_O and CD_3_OD was used as the solvent for NMR hydrogen spectroscopy. Moreover, a series of oHA-ss-PTX with different PTX loading capacity was synthesized by varying the dosage ratio of 2′sPTX to oHA-ss-NH_2_ (7/20, 10/20, 13/20, 16/20, 20/20).

### 2.3. Preparation of oHA-ss-PTX Micelles and ADM/oHA-ss-PTX Micelles

ADM hydrochloride was dissolved in a methanol solution. Then, the solution was slowly dropped into twice the molar mass of triethylamine at the same time. The mixture was stirred in a 37 °C water bath for 24 h and protected from light. The desalted ADM was dissolved in 1.5 mL N, N-dimethylformamide for subsequent use in micelles preparation. ADM/oHA-ss-PTX micelles were prepared in one step. oHA-ss-PTX and ADM were dissolved in ethanol, at a volume ratio of 3:1 (PBS: ethanol) and flow rate of 4~12 mL/min using a microfluidic mixer (Inano L, Micro&Nano Technology Inc., Suzhou, China). Then, the obtained micelles were dialyzed in 2 kDa membrane dialysis cassettes against phosphate-buffered saline (PBS, pH 7.4) for at least 24 h, the dialyzed micelles were concentrated using 3 kDa ultracentrifugal filters.

### 2.4. Characterization of oHA-ss-PTX Micelles and ADM/oHA-ss-PTX Micelles

The particle size distribution and zeta potential were detected using a nanoparticle size analyzer (Litesizer 500, Anton Paar, Graz, Austria). The particle size of 1 mL micelle solution (0.5 mg/mL) was detected by Dynamic Light Scattering (DLS). Transmission electron microscopy (TEM) measurements were performed on a transmission electron microscope with an accelerating voltage of 100 kV. A drop of micelle solution (0.1 mg/mL) was deposited onto a 230 mesh carbon-coated copper grid and allowed to dry in air at 25 °C before measurements. The critical micellar concentration (CMC) of oHA-ss-PTX micelles was measured using pyrene as a fluorescence probe. The fluorescence spectra of pyrene were measured by a FLS920 fluorescence spectrometer with an excitation wavelength of 330 nm. The CMC was estimated at the cross-point obtained by extrapolating the intensity ratio I_339_/I_334_ at low- and high-concentration regions.

### 2.5. Loading Content and Encapsulation Efficiency of Micelles

The content of ADM in the ADM/oHA-ss-PTX micelles was measured by UV–Vis Spectrophotometer-2700 and the detection wavelength set at 485 nm. And the content of PTX in the oHA-ss-PTX conjugate was evaluated by HPLC, the detection wavelength was 227 nm. The entrapment efficiency (EE%) and loading content (LC%) were calculated according to the following equation:

EE% = (Weight of drug in micelles)/(Weight of feeding drug) × 100%(1)

LC% = (Weight of drug in micelles)/(Weight of micelles) × 100%(2)

### 2.6. Drugs Release of Micelles by Glutathione In Vitro

The release profiles of PTX and ADM from ADM/oHA-ss-PTX were examined by dynamic dialysis method. First, 1 mL of ADM/oHA-ss-PTX micelles solution was placed into a dialysis bag (MWCO: 2 kDa). Subsequently, the dialysis bag was immersed in 25 mL release media, consisting of 0.5% (*w*/*v*) Tween 80, 10 mM GSH and PBS, Then, the sample was gently shaken at 37 °C in constant temperature shaking incubator at 100 rpm. At the prospective time intervals, 8 mL release media was collected and the whole old media was replaced by fresh media. The drugs were released from ADM/oHA-ss-PTX micelles in media without GSH as the control. The amount of ADM was measured (UV–Vis Spectrophotometer-2700, detection wavelength 485 nm) and PTX was measured by HPLC (chromatographic column Diamonsil C18, mobile-phase ratio acetonitrile: water 60:40, flow rate 1.0 mL/min, detection wavelength 227 nm and peak time at 7.04 min).

### 2.7. Toxicity of oHA-ss-NH_2_ and ADM/oHA-ss-PTX to ECs

ECs were invoked as a model to evaluate the cytotoxicity of oHA-ss-NH_2_ and ADM/oHA-ss-PTX via CCK8 method. First, 5 × 10^3^ ECs per well were seeded in a 96-well plate with five replicate wells. Then, the medium was used to prepare different concentrations of oHA-ss-NH_2_ (25 μg/mL, 50 μg/mL, 100 μg/mL, 200 μg/mL, 400 μg/mL, 600 μg/mL) and ADM/oHA-ss-PTX (17 μM, 34 μM, 68 μM, 136 μM, 227 μM). And free ADM and PTX were used at an equal dose according to the drug loading amount in the micelles. After 24 h of incubation, the culture medium in the 96-well plate was removed. Next, 200 μL of complete medium containing different concentrations of oHA-ss-NH_2_ and ADM/oHA-ss-PTX was added, respectively. The wells without oHA-ss-NH_2_ and ADM/oHA-ss-PTX were used as the control. The group without cells was used as black. After another 24 h incubation, the inhibition rate or the survival rate of oHA-ss-NH_2_ and ADM/oHA-ss-PTX was evaluated. The cell survival rate was then calculated according to the following formula:

Cell viability (%) = (A450_Drugs_ − A490_Blank_)/(A450_Control_ − A490_Blank_) × 100% (3)

### 2.8. Hemolysis Testing

The 2% red blood cell suspension, prepared by diluting 2 mL of red blood cells from New Zealand white rabbits to 100 mL, was invoked as the experimental model of hemolysis. First, 0.5 mL of different concentrations of ADM/oHA-ss-PTX and free drugs (PTX+ADM) were placed in tubes numbered 1–5 and A-E, respectively. Tubes No. 6 and No. 7 were designated as negative and positive controls, respectively. Next, 2% red suspension (2.5 mL) and normal saline (2.0 mL) were added to tubes 1–5 and A-E, respectively. In addition, 2% red suspension (2.5 mL) and double distilled water (2.5 mL) were added to No. 6 and No. 7. Take out the test tube and centrifuge for 15 min at 3000 r/min after incubating at 37 °C for 3 h. PBS and distilled water were used as negative and positive controls, respectively. UV–Vis spectrophotometer was used to detect the absorbance of the sample supernatant at 540 nm, and the Hemolysis Rate (HR) was calculated according to the following equation:

HR (%) = (A_sample_ − A_negative_)/(A_positive_/A_negative_) × 100% (4)

### 2.9. Bovine Serum Albumin (BSA) Adsorption

The BSA adsorption of ADM/oHA-ss-PTX micelles was evaluated by the Bradford method. First, 4 mL of BSA solution (400 μg/mL) was added to 11 tubes (numbered 1~6 and A-E), then 500 μL of different concentrations of ADM/oHA-ss-PTX and free-drug (PTX+ADM) solutions were added to tubes numbered 1–5 and A-E, respectively. For tube 6, 500 μL of normal saline was added as a blank control. All the tubes were placed in a constant temperature oven at 37 °C after they were uniformly mixed. The samples were taken out and placed in an ultra-high-speed centrifuge at 12,000 r/min for 15 min after being incubated for 2 h, 4 h, and 6 h. After centrifugation, 100 μL of the supernatant was added to 4 mL of G-250 dye solution. The mixture was vortexed to ensure thorough mixing and then allowed to stand for 3 min. The samples were detected at 595 nm in the UV–Vis spectrophotometer, and the protein adsorption rate was calculated according to the following equation:

Adsorption% = (A_control_ − A_sample_)/A_control_ × 100% (5)

### 2.10. Evaluation of Anticancer Effect In Vitro

MCF-7 cells and A549 cells were used to evaluate the in vitro anticancer effect of ADM/oHA-ss-PTX micelles by CCK8 method and compared with the free drug. The drug concentrations of ADM/oHA-ss-PTX micelles were 100 μM, 50 μM, 10 μM, 5 μM, and 1 μM, respectively. And free ADM and PTX were used at an equal dose according to the drug loading amount in the micelles. The wells without oHA-ss-NH_2_ and ADM/oHA-ss-PTX was used as control. The wells without cells was used as black. The time of drug treatment of the cells was 48 h. The cell survival rate was then calculated according to Formula (3). The cell inhibition rate was calculated according to the following equation:

Cell inhibition (%) = 100% − Cell viability (%)(6)

### 2.11. Evaluation of Synergistic Antitumor Effect In Vitro

SPSS Statistics 24 software was used to calculate IC_50_ and all values of PTX, ADM, ADM/oHA-ss-PTX, (ADM+PTX) ranging from IC_20_~IC_90_. Then, the Combination Index (CI) was calculated according to the following equation:

CI = D_A_/(Dx)_A_ + D_B_/(D_X_)_B_ (7)

where (Dx)_A_ and (Dx)_B_ represent the ICx values of the free drugs PTX and ADM alone. D_A_ and D_B_ represent the ICx values of PTX and ADM in the mixed drug group or the micellar group.

### 2.12. Cellular Internalization on Targeting of ADM/oHA-ss-PTX

A549 cells were used to evaluate the CD44 targeting of ADM/oHA-ss-PTX micelles by fluorescence microscopy. First, A549 cells were seeded at a density of 5 × 10^4^ cells/well in six-well plate. The experiment was divided into four groups, ADM+PTX, (ADM+PTX)+oHA, ADM/oHA-ss-PTX, (ADM/oHA-ss-PTX)+oHA. In the groups containing oHA, 1 mg/mL oHA was added and the cells incubated for 2 h to block CD44 on the surface of A549 cells. The 6-well plate was removed and the medium was immediately aspirated and the 6-well plate was washed twice with cold PBS buffer after incubating with the drug for 2 h. Next, approximately 1 mL of 4% paraformaldehyde solution was added to fix the cells (10 min) after the remaining PBS solution in the 6-well plate was exhausted. Subsequently, 0.5 mL of DAPI solution was added to the 6-well plate washed with cold PBS to stain the nuclei in the dark for 15 min. The multifunctional inverted fluorescence microscope was immediately utilized to observe the drug entering the cells (ADM was red, DAPI-stained nucleus was blue) when the nucleus was stained. And Image J 1.54P software was utilized to analyze data.

### 2.13. In Vitro Migration and Invasion

A549 cells were seeded in 6-well plates, then cells were wounded with a p200 pipette tip and then treated with control, ADM, PTX, ADM+PTX, oHA-ss-PTX and ADM/oHA-ss-PTX for 24 h. The area of wound healing was measured under a microscope (Olympus, Tokyo, Japan). As for invasion assays, the number of cells tested was 2 × 10^5^. After treatment for 48 h, non-migrated and non-invaded cells in the upper wells were removed with a cotton swab. The cells that had passed through the membrane and adhered to the lower surface were fixed with 75% ethanol, stained with crystal violet, and then quantified under a microscope.

### 2.14. In Vivo Biodistribution of Micelles

The C57BL/6 mice (6 weeks, 18–22 g) were subcutaneously injected into the armpit of right anterior limb with 0.10 mL of cell suspension containing 2.0 × 10^6^ LLC cells in cell culture medium. After the tumor in the mice reached a volume of approximately 150 mm^3^, the C57BL/6 mice were injected via the tail vein with ADM/oHA-ss-PTX and free ADM. At different time points after injection, the fluorescent signals of ADM in animals were measured using an imaging system (PE IVIS Spectrum, Waltham, MA, USA).

### 2.15. In Vivo Antitumor Efficacy

The male C57BL/6 (6 weeks,18–22 g) mice with LLC cells implanted in the armpit of the right anterior limb were used to evaluate the in vivo antitumor efficacy of micelles. The mice were injected subcutaneously in the armpit of right anterior limb with 0.10 mL of cell suspension containing 2.0 × 10^6^ LLC cells in cell culture medium. The mice were weighed and randomly divided into 3 groups (6 mice per group), when the tumors in the mice reached a volume of approximately 150 mm^3^. Then, the treatments were initiated and were designated as day 0, 2, 4, 6, 8, 10, 12, and 14, respectively. The mice in different groups were given tail-vein injections of free ADM/PTX and ADM/oHA-ss-PTX, saline as the blank control. One days after the last treatment, mice were sacrificed, and blood and organs were collected for further analysis. Tumor volumes were calculated according to the following equation:

V (mm^3^) = (L × W^2^)/2 (8)

### 2.16. Organ Damage Assays

The mice were weighed and randomly divided into 3 groups (6 mice per group). Two groups were, respectively, administered free ADM+PTX and ADM/oHA-ss-PTX, while the remaining group received saline as the blank control. Right after the last treatment, the blood of each mouse was collected into a coagulation-promoting tube. Subsequently, the blood samples were centrifuged at 4000 rpm for 10 min to obtain plasma for the measurement of clinical parameters. In this experiment, several clinical parameters were detected, including alanine aminotransferase (ALT), aspartate aminotransferase (AST), blood urea nitrogen (BUN), creatinine (Cr), creatine kinase (CK), creatine kinase-MB (CK-MB) and lactate dehydrogenase (LDH) were detected [29,30]. As is known to all, these parameters are closely related to the function or potential organ damage of the liver (ALT, AST), heart (CK, CK-MB and LDH) and kidney (BUN and Cr).

### 2.17. Statistical Analysis

The data were reported as the mean ± standard deviation. Student’s *t*-test was used to determine the statistical difference comparisons between two groups. One-way analysis of variance (ANOVA) was used to analyze the statistical difference among multiple group comparisons. Differences were considered statistically significant at a level of *p* ≤ 0.05. In figures, levels of significance are denoted as follows: *p* < 0.05 (*), *p* < 0.01 (**), and *p* < 0.001 (***).

## 3. Results

### 3.1. Synthesis and Characterization of oHA-ss-PTX

#### 3.1.1. Analysis of FTIR and ^1^HNMR of oHA-ss-NH_2_

As shown in Figure S1, the characteristic bands of oHA were observed at 1153 cm^−1^, 1083 cm^−1^, 1043 cm^−1^, and 947 cm^−1^. In the FTIR spectrum of oHA, the stretching vibration at 3421 cm^−1^ was assigned to the -OH stretching vibration. In the FTIR spectrum of oHA-ss-NH_2_, the stretching vibration peak that was initially at 3421 cm^−1^ shifted to 3564 cm^−1^, which may be associated with the -NH stretching vibration of the primary amine. Notably, in the ^1^H-NMR spectrum of oHA, the chemical shift range of 3.30–4.53 ppm was attributed to the glycoside protons within the oHA repeat unit, and the signal at 2.01 ppm was assigned to the acetyl methyl proton. When comparing the ^1^H-NMR spectrum of oHA-ss-NH_2_ with that of oHA (Figure S2), a new proton signal emerged at 2.82 ppm. Therefore, combined with the FTIR, it can be fully proved that cystamine was successfully grafted onto oHA, and oHA-ss-NH_2_ was synthesized.

#### 3.1.2. Analysis of FTIR and ^1^HNMR of 2′sPTX

The FTIR spectra of PTX and 2′sPTX are shown in Figure S3. In these spectra, the peaks at 1732 cm^−1^ and 1714 cm^−1^ can be attributed to the C=O stretching vibration of the ester bond and the ketone group, respectively. The sharp peak observed at 1724 cm^−1^ is considered to be the result of the overlap between the C=O stretching vibration of succinate, which was introduced after the modification with succinic anhydride, and the C=O stretching vibration of PTX itself. In addition, the absorption band at 2926 cm^−1^ is assigned to the stretching vibration of -CH_2_ groups in succinic acid. Crucially, the characteristic aromatic proton resonance signal appears at the chemical shift range of 7.32–8.12 ppm in the ^1^HNMR of 2′sPTX (Figure S4). Moreover, the proton resonance signal at 2.32–2.61 ppm is attributed to -CH_2_- groups within succinic anhydride moiety. Undoubtedly, the combined FTIR results indicate that the connection of succinic anhydride was successful.

#### 3.1.3. Analysis of FTIR and ^1^HNMR of oHA-ss-PTX

**Figure 1:**
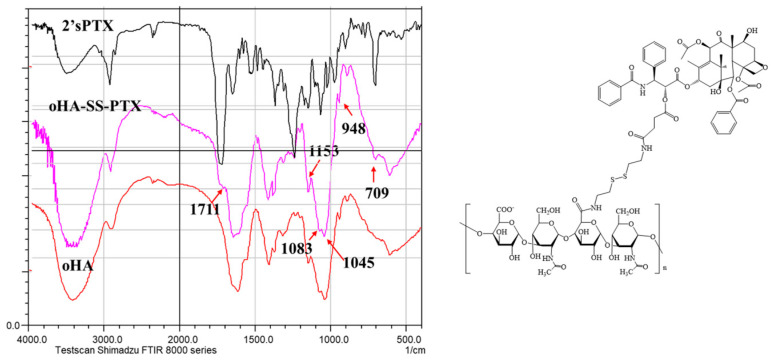
FT-IR spectra characterization of oHA-ss-PTX.

**Figure 2:**
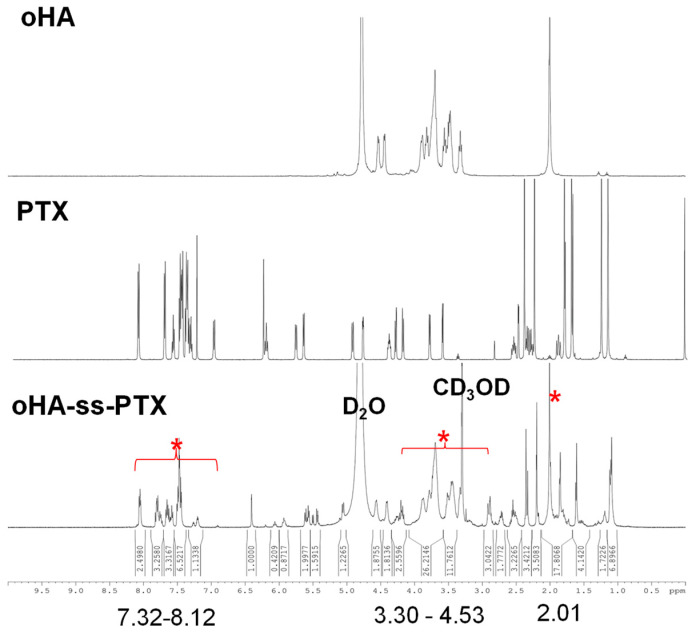
^1^H-NMR characterization of oHA-ss-PTX (* characteristic chemical shift).

The infrared spectrum of oHA-ss-PTX is shown in Figure 1. In this spectrum, the absorption bands at 1153 cm^−1^, 1083 cm^−1^, and 1045 cm^−1^ can be assigned to the characteristic bands of oHA. The absorption at 709 cm^−1^ is considered to be associated with the substituted benzene of PTX. In addition, the absorption peak at 1711 cm^−1^ is linked to the C=O stretching vibration in 2′sPTX. In the ^1^HNMR spectrum of oHA-ss-PTX (Figure 2), a significant aromatic proton resonance signal is observed in the chemical shift range of 7.32–8.10 ppm, which was consistent with the characteristic signal of PTX. Additionally, the proton resonance signal at 3.32–4.55 ppm and 2.02 ppm can be attributed to the glycoside protons and acetyl methyl protons in the repeating unit of oHA, respectively. Based on the relevant information from both the infrared spectrum and the ^1^H-NMR spectrum, it can be concluded that PTX was successfully combined with oHA.

### 3.2. Characterization of Micelles

#### 3.2.1. Preparation Process Screening of Prodrug oHA-ss-PTX Micelles

The drug LC% and EE% of PTX and ADM were measured by HPLC and UV–Vis spectrophotometry [18]. The results showed that with the increase in the dosage ratio of 2′sPTX and oHA-ss-NH_2_ (7/20, 10/20, 13/20, 16/20), the LC% of PTX increased from 4.8 ± 0.2% to 22.8 ± 1.6% (Table S1). And the particle size of oHA-ss-PTX micelles decreased from 174 ± 1.2 nm to 107 ± 2.4 nm as the amount of PTX grafted increased. Meanwhile, the LC% of ADM also showed an upward trend with the increase in the grafting amount of PTX. The maximum LC% of ADM was 7.6 ± 0.2% and the EE% was nearly 82.3 ± 4.7%, as shown in Table S2. However, a high drug loading of PTX led to a significant decrease in the water solubility of the oHA-ss-PTX conjugate. When the dosage ratio to the 20/20, a slight precipitation appears in the micelles solution. Therefore, we selected 16/20 for the subsequent experiments.

#### 3.2.2. Physicochemical Characteristics of the ADM/oHA-ss-PTX Micelles

**Figure 3:**
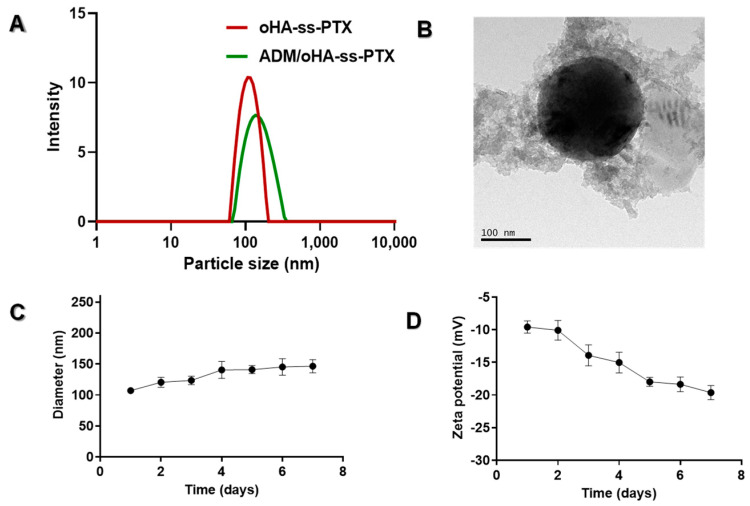
Physicochemical characteristics of ADM/oHA-ss-PTX. (**A**) size distribution; (**B**) TEM image; (**C**) Particle size at different time; (**D**) ζ potential at different time.

We prepared ADM/oHA-ss-PTX by a microfluidic process. Experimental data showed that LC% of PTX was 21.2 ± 2.3% and LC% of ADM was 7.6 ± 0.2%, as shown in Table S2. The particle size presents a normal distribution (PDI = 0.13 ± 0.038), the average particle size is approximately 127 ± 1.4 nm. In Figure 3B, the particle size observed in TEM images was slightly smaller than the hydrodynamic diameter measured. When compared with oHA-ss-PTX, the particle size of ADM/oHA-ss-PTX has increased, as depicted in Figure 3A,B and Tables S1 and S2. It is likely that ADM is physically embedded within the micelles, causing the inner core of the micelles to enlarge. The reduction in the grafting amount of PTX leads to a decrease in the hydrophobic interaction force within the hydrophobic core of the micelle, which is also considered to be the possible reason for the increase in the micelle’s particle size [31,32]. The zeta potential of oHA-ss-PTX was −20.9 mV compared to other oHA-ss-PTX with different drug loadings. However, a small portion of ADM was adsorbed on the surface of the negatively charged oHA-ss-PTX micelles, resulting in a decrease in the zeta potential of ADM/oHA-ss-PTX (−9.0 mV), as shown in Tables S1 and S2. As for the microfluidics preparation process, a flow rate of 4 mL/min was determined to be the optimal value.

#### 3.2.3. Stability Evaluation of ADM/oHA-ss-PTX Micelles

As shown in Figure 3C,D, the particle size of ADM/oHA-ss-PTX micelles can remain relatively stable in double distilled water within 7 days. After the 7 days, the particle size of the micelles still less than 150 nm. In addition, the ζ potential of micelles fluctuated slightly for 7 days. This might be due to the detachment of ADM adsorbed on the surface of the micelles, which is consistent with the decrease in zeta potential after encapsulating ADM as mentioned before. The micelle solution remained clear after one month. This indicated that the micelles have a certain degree of stability, and it also lays a theoretical foundation for the drug delivery of micelles.

**Figure 4:**
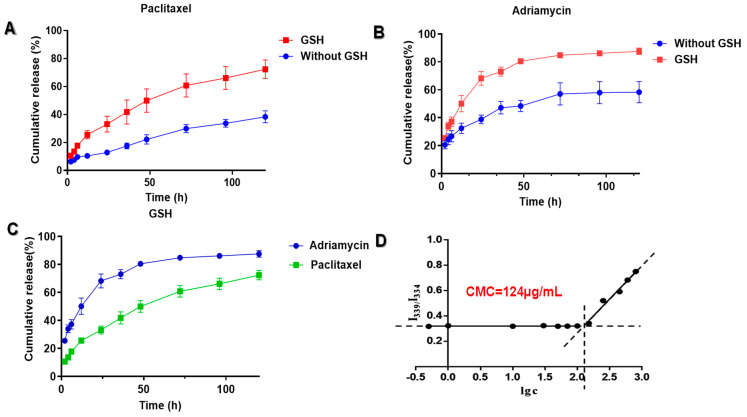
In vitro release experiments of ADM/oHA-ss-PTX and CMC testing. (**A**) PTX release from ADM/oHA-ss-PTX; (**B**) ADM release from ADM/oHA-ss-PTX; (**C**) the superposition curves of the release of PTX and ADM under GSH stimulation; (**D**) quotient of vibrational band intensities (I_339_/I_334_) from excitation spectra of pyrene as a function of lgc of oHA-ss-PTX.

As shown in Figure 4D, the experimental results showed that the CMC of micelles is 124 μg/mL. The lower CMC reflects the excellent antidilution ability of oHA-ss-PTX, which can effectively resist the dilution from the blood, thus facilitating the stable delivery of the drug in the systemic circulation.

### 3.3. In Vitro Drug Release of ADM/oHA-ss-PTX Micelles

The cumulative release curve of PTX was shown in Figure 4A. As is evident from the figure, under the stimulation of GSH, the cumulative release of PTX reaches 49.9% within 48 h. However, PTX was released only 25.0% within 48 h when there was no GSH trigger. The cumulative release curve of ADM is shown in Figure 4B. The results showed that ADM is released relatively slowly in the GSH-free medium, with only 48.4% of the drug being released after 48 h. However, in the GSH-containing medium, ADM is released at a much faster rate. Specifically, at 48 h and 120 h, the release rates of ADM are 80.5% and 87.5%, respectively. Furthermore, as can be observed from Figure 4C, under the same GSH-containing medium, the release rate of PTX is slower than that of ADM. The emergence of this result lays a solid foundation for the co-loaded micelles to achieve the goals of synergistic antitumor effects and maintenance therapy.

### 3.4. Biocompatibility of ADM/oHA-ss-PTX Micelles

#### 3.4.1. Cytotoxicity of oHA-ss-NH_2_ and ADM/oHA-ss-PTX

**Figure 5:**
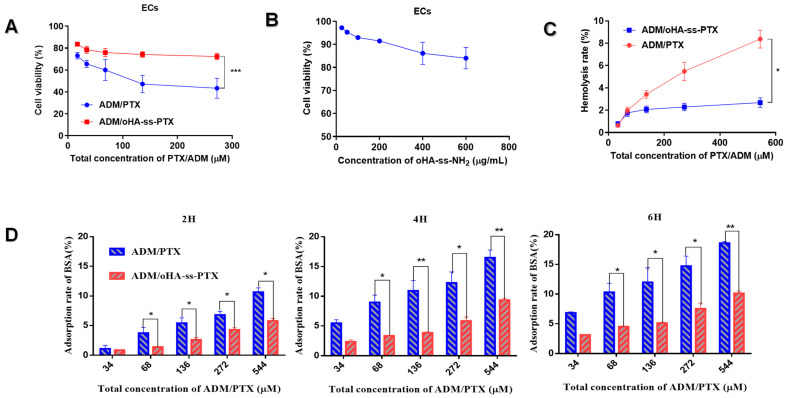
Biocompatibilities evolution. (**A**) ADM/oHA-ss-PTX micelles and free ADM+PTX on the cell viability of ECs cells(n = 3, *** *p* < 0.001); (**B**) oHA-ss-NH_2_ on the cell viability of ECs cells; (**C**) Percentage of RBCs hemolysis incubated with ADM/oHA-ss-PTX micelles and free ADM+PTX (n = 3, * *p* < 0.05); (**D**) BSA adsorption on ADM/oHA-ss-PTX micelles and free ADM+PTX after incubation at 37 °C, different concentrations and periods of time: 2, 4 and 6 h (n = 3, * *p* < 0.05, ** *p* < 0.01).

To investigate the cell biocompatibility of ADM/oHA-ss-PTX, the proliferation inhibition of normal cells (ECs) was evaluated by a CCK-8 assay. As shown in Figure 5B, even when the concentration of oHA-ss-NH_2_ reached 600 μg/mL, the survival rate of ECs still exceeded 80%. This result strongly suggests that that oHA-ss-NH_2_ materials possess excellent biocompatibility. It can be seen from Figure 5B that although ADM/oHA-ss-PTX micelles at high concentrations (272 μM) have a killing effect on ECs (inhibition ratio 29.2%), when compared with the free-drug group (inhibition ratio 58.7%), the toxicity of ADM/oHA-ss-PTX micelles is significantly milder (*p* < 0.001) (Figure 5A). This indicated that the ADM/oHA-ss-PTX micelles can effectively reduce the toxicity of the PTX and ADM.

#### 3.4.2. Hemolysis Rate of ADM/oHA-ss-PTX Micelles

The hemolysis rate of ADM/oHA-ss-PTX micelles at each concentration is lower than 5% (hemolysis rate > 5% is regarded as hemolysis). However, hemolysis occurred when the concentration of the free-drug group exceeded 136 μM. and hemolysis was more significant as the concentration increased (*p* < 0.05), as shown in Figure 5C. This suggests that micelles can effectively reduce the hemolysis rate of the drug, and minimize the potential hemolysis that could occur when the drug enters the bloodstream.

#### 3.4.3. BSA Adsorption of ADM/oHA-ss-PTX Micelles

The BSA adsorption rate of ADM/oHA-ss-PTX micelles and free drugs increased with time and drug concentration. However, the protein adsorption rate of ADM/oHA-ss-PTX micelles (9.4%) was significantly lower than that of free drug (16.8%), and this discrepancy became more pronounced with the increase in time and drug concentration (*p* < 0.01), as shown in Figure 5D. This also fully reflected the remarkable biocompatibility of ADM/oHA-ss-PTX micelles.

### 3.5. Cellular Proliferation Inhibitions and Cell Apoptosis

**Figure 6:**
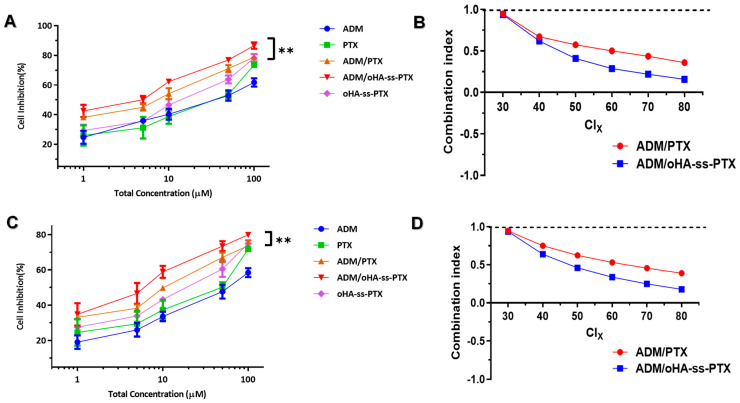
In vitro cellular assay. (**A**) Cell inhibition of A549 cells for 48 h (n = 3, ** *p* < 0.01); (**B**) Combination Index in A549 cells; (**C**) cell inhibition of MCF-7 cells for 48 h (n = 3, ** *p* < 0.01); (**D**) Combination Index in MCF-7 cells.

The proliferation inhibitions of A549 cells and MCF-7 cells were evaluated by CCK-8 assay (Figure 6A,C). All the formulations, including free ADM+PTX, oHA-ss-PTX, ADM, PTX, and ADM/oHA-ss-PTX, show both time- and dose-dependent proliferation inhibition effects. Compared to the free ADM+PTX formulation, the ADM/oHA-ss-PTX micelles exhibited a significantly greater inhibitory activity on the proliferation of A549 cells and MCF-7 cells, which is likely attributed to the co-delivery of ADM and PTX by the ADM/oHA-ss-PTX micelles for enhanced therapeutic efficacy of the combination of ADM+PTX. Surprisingly, ADM/oHA-ss-PTX exhibited a more significant inhibitory activity on the proliferation of A549 cells. This may be related to the rate of cell proliferation due to cell resistance [33,34]. Based on this, we selected lung cancer cells for subsequent experiments.

The Combination Index (CI) indicates that the drugs have a synergistic effect when CI < 1, CI = 1 indicates that the drugs have an additive effect and CI > 1 indicates that the drugs have an antagonistic effect [35,36]. The CI of the ADM+PTX mixture and ADM/oHA-ss-PTX micelles was less than 1 within the range from CI_30_ to CI_80_. This result suggested that whether it was free ADM+PTX or ADM/oHA-ss-PTX micelles, both can produce effective synergistic antitumor effects. Moreover, the synergistic effect of ADM/oHA-ss-PTX micelles was more prominent than the ADM+PTX group, as shown in Figure 6B,D.

### 3.6. Cellular Internalization

**Figure 7:**
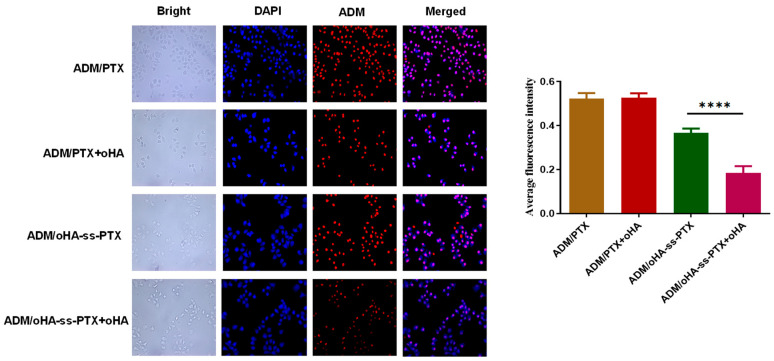
Cell uptake. Fluorescence microscope images (100×) and quantification of A549 cells (n = 5, **** *p* < 0.0001).

**Figure 8:**
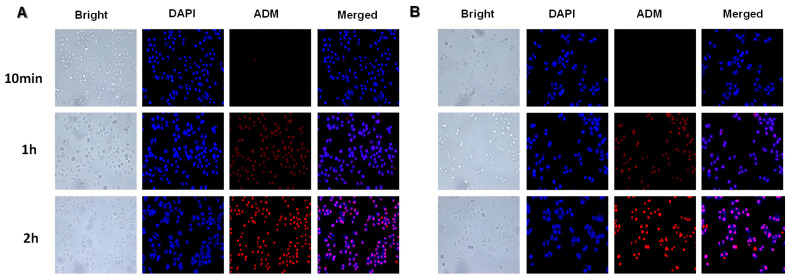
Cell uptake for different time. Fluorescence microscope images (100×, (**A**) ADM+PTX; (**B**) ADM/oHA-ss-PTX) of A549 cells for 10 min, 1 h and 2 h.

The targeting ability of ADM/oHA-ss-PTX micelles was evaluated through a competitive antagonism experiment, as shown in Figure 7. The results of this study showed that A549 cells saturated with oHA had lower ADM/oHA-ss-PTX micelle uptake than A549 cells without oHA treatment (*p* < 0.05). However, there is no statistical difference in the uptake of free ADM+PTX by A549 cells saturated or unsaturated with oHA. The occurrence of this phenomenon was related to the inhibition of CD44-mediated endocytosis by oHA targeting CD44. As time elapsed, the amount of ADM uptake by A549 cells increased gradually (Figure 8).

### 3.7. In Vitro Antimetastatic Effects

**Figure 9:**
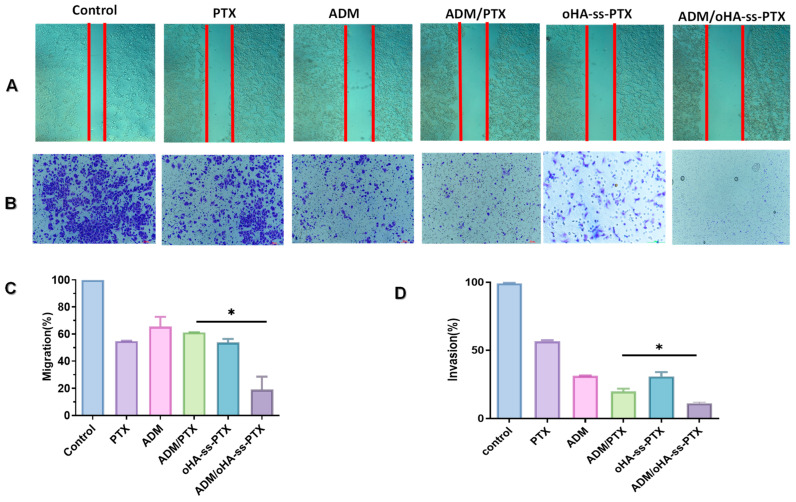
In vitro antimetastatic effects. (**A**) The wound healing images (40×) and (**C**) quantitative analysis (n = 5, * *p* < 0.05) after scratch for 24 h. (**B**) Microscopy images (100×) of invasion (**D**) and quantitative analysis (n = 5, * *p* < 0.05).

As demonstrated in Figure 9A,C, the wound healing assay was carried out to evaluate the inhibition ability of micelles on cell motility and interactions. The migration of A549 cells were effectively inhibited by PTX, ADM, (ADM+PTX), oHA-ss-PTX, ADM/oHA-ss-PTX. Among them, the inhibitory effect of ADM/oHA-ss-PTX was more prominent than ADM+PTX (* *p* < 0.05). Which indicated that ADM/oHA-ss-PTX not only effectively inhibited tumor cell growth but also significantly reduced the tumor cell migration rate.

The invasion experiment was carried out to further estimate the effects of micelles on cellular motility ability of A549 cells. The control group (without any agents) showed the high invasion ability, which was consistent with the manifestation of wound healing assay (Figure 9B). All the tested agents demonstrated inhibitory effects on cellular motility of A549 cells. The PTX, ADM, (ADM+PTX), oHA-ss-PTX, ADM/oHA-ss-PTX displayed a certain degree of inhibition on invasion of A549 cells with invasion rates at 56.64%, 31.31%, 19.91%, 30.91% and 11.14%, respectively. But the influences of ADM/oHA-ss-PTX on cell invasion were more remarkable than that of ADM+PTX. The results showed better inhibitory effect of ADM/oHA-ss-PTX.

### 3.8. In Vivo Fluorescence Imaging

**Figure 10:**
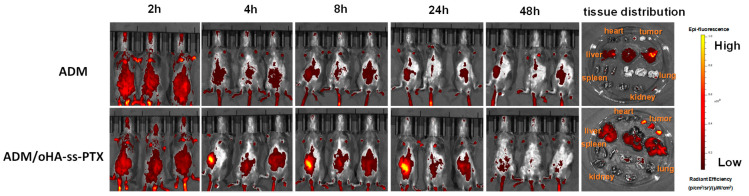
In vivo fluorescence imaging.

Significant differences in the ADM fluorescence signals were observed in tumor-bearing mice at different time points following intravenous injection of ADM/oHA-ss-PTX. Specifically, the fluorescence intensity of ADM in the tumor sites of mice continuously increased over time and peaked at 24 h post-injection (Figure 10). Subsequently, the fluorescence signal gradually weakened. Therefore, tumor tissues and main organs of nude mice were collected at 24 h post-injection. When comparing the fluorescence signals, it was evident that ADM/oHA-ss-PTX micelles had a greater accumulation at the tumor site compared with free ADM. This observation suggests that ADM/oHA-ss-PTX micelles possess good targeting ability. Overall, ADM/oHA-ss-PTX micelles demonstrated excellent tumor accumulation ability, which could potentially enhance the targeted therapy of cancer.

### 3.9. In Vivo Antitumor Efficacies

**Figure 11:**
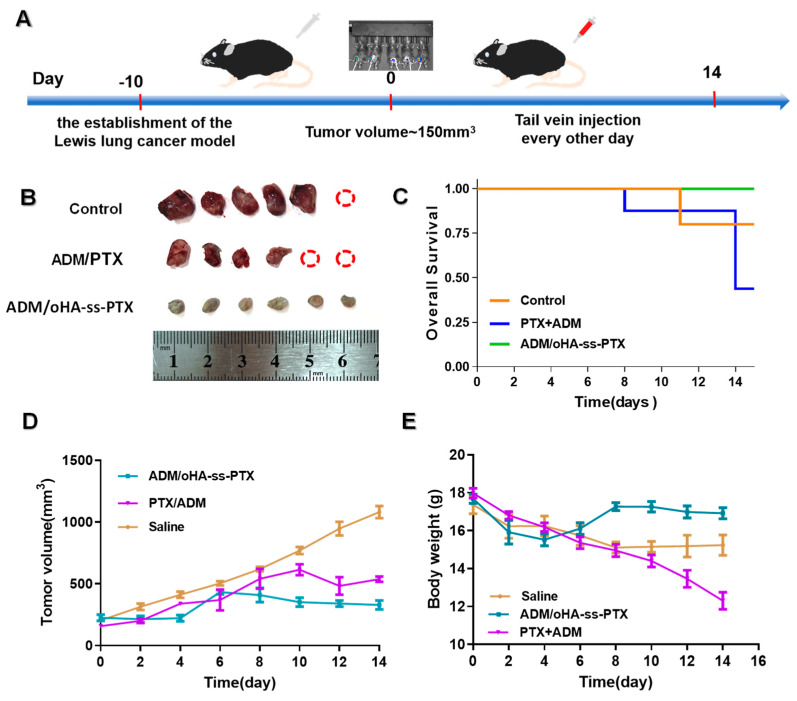
In vivo antitumor efficacies. (**A**) Diagram of animal experiment procedure; (**B**) representative photos of tumor (the red dotted line: the mice that died during the experiment process); (**C**) the survival situation of animals; (**D**) inhibition of tumor growth after different treatments; (**E**) body weights of mice with various treatments for the 14-day evaluation period.

The antitumor efficacy of micelles in vivo were evaluated using a LLC cells xenograft mice model. LLC cells were injected into the armpit of right anterior limb of mice (Figure 11A). When the tumors reached a volume of more than 150 mm^3^, in approximately 10 days after inoculation of the cells, the mice were randomly divided into 3 groups, with 6 mice in each group. Treatments were administered on day 0, 2, 4, 6, 8, 10, 12, 14. The formulations for the three groups were as follows: saline, free ADM+PTX, ADM/oHA-ss-PTX. The tumor volume (Figure 11B,D) and body weight (Figure 11E) were later monitored over a treatment period of 14 days and the results revealed that ADM/oHA-ss-PTX micelles were more effective formulation to suppress the tumor growth as compared to ADM/PTX (Figure 11B).

As shown in Figure 11C, throughout the treatment period, no mortality was observed in the group treated with ADM/oHA-ss-PTX micelles. In contrast, two mice in the ADM+PTX group succumbed. This finding further demonstrated that the ADM/oHA-ss-PTX micelles possess the capacity to mitigate the toxicity. In Figure 11E, insignificant body weight losses were observed after the administration of micelles compared to the initial body weights of tumor-bearing mice during the whole experimental period, indicating that micelles were well tolerated at the tested dosage level. While mice treated with free ADM+PTX at the same dosage level showed weight loss, this may be due to the toxicity of ADM+PTX.

**Figure 12:**
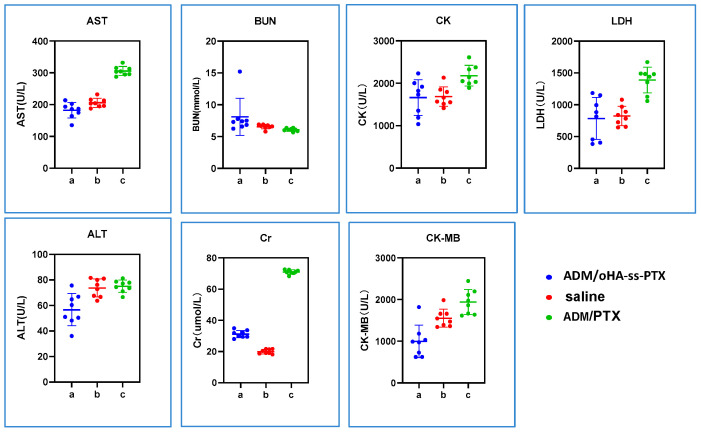
Toxicity and side-effect evaluation. Testing ALT, AST, BUN, Cr, CK, CK-MB and LDH levels after all the treatments.

In order to compare the systematic toxicities of free ADM+PTX and ADM/oHA-ss-PTX micelles after all treatments, alterations of clinical chemical parameters in mice after drug treatment were determined, including ALT, AST, BUN, Cr, CK, CK-MB and LDH. It is well known that these parameters are associated with the function or potential damage of liver (ALT, AST), kidney (BUN and Cr) and heart (CK, CK-MB and LDH). As shown in Figure 12, the values of these parameters of ADM/oHA-ss-PTX substantially maintained at normal levels, but the parameters of ADM+PTX fluctuated greatly: the level of AST, Cr, CK and LDH was significantly higher, indicating that the tail-vein injection of ADM+PTX causes damage to the liver, kidney, and heart of mice in the treatment duration.

## 4. Discussion

HA has already achieved remarkable results in the delivery of antitumor drugs [37,38]. In contrast to HA, oHA not only retains excellent biocompatibility and biodegradability, but also exhibits an elevated higher cell-targeting proficiency and drug-encapsulation efficiency. In addition, oHA demonstrates favorable water solubility. The design of drug delivery systems modified by disulfide bonds (-ss-) holds paramount significance in counteracting the challenges posed by the tumor microenvironment (TME), which is typified by conspicuously elevated GSH levels (approximately 10 mM in contrast to around 2 μM in normal tissues) [26]. Disulfide bonds function as redox-responsive “control switches”, maintaining stability during systemic circulation under conditions of low GSH concentration, yet promptly cleaving upon encountering the TME, thereby facilitating tumor-specific drug release and effectively curtailing off-target toxicity [39,40]. This innovative strategy augments therapeutic precision by integrating controlled release mechanisms with active targeting ligands, such as oHA and folic acid (FA), which are conducive to enhanced tumor accumulation [41,42]. Moreover, disulfide linkages contribute to bolstering the systemic stability of the drug delivery system, precluding premature drug leakage and concurrently synergizing with other stimuli-responsive modalities, like pH and enzyme-responsive elements, for orchestrated and cascaded drug activation. Hence, we selected oHA as the carrier material and disulfide bond(-ss-) as the linker for the connection between PTX and oHA.

Microfluidic technology plays a pivotal role in micelle fabrication. This technology facilitates swift and uniform blending of amphiphilic constituents, which in turn fosters stable self-assembly processes while curtailing the residual amounts of organic solvents to a minimum [43,44]. The scalable nature of its design smoothens the path from laboratory-scale optimization to industrial-scale manufacture, as exemplified by the production of lipid nanoparticles for mRNA vaccines [45,46]. In contrast to traditional approaches, microfluidics not only augments drug-encapsulation efficiency to over 90% but also permits the construction of intricate architectures, such as core-shell structures, for targeted drug delivery. Its remarkable reproducibility and adaptability establish it as an indispensable tool for propelling the development of nanocarrier systems in the realm of precision medicine. In this work, microfluidic technology was used to prepare amphiphilic micelles and co-delivery of ADM.

In this study, the oHA-ss-PTX macromolecular prodrug was synthesized by introducing GSH-stimuli-responsive disulfide bonds through chemical reactions. Briefly, oHA-ss-NH_2_ was synthesized based on conjugation of cystamine to oHA by an amide bond. And PTX was successfully carboxylated (2′sPTX) through an esterification reaction. Subsequently, the oHA-ss-PTX was synthesized via an amidation reaction. Then, a microfluidic process was used to prepare ADM/oHA-ss-PTX micelles in one step. We obtained a kind of nanomicelle with a high drug loading capacity and a small size. The delivery efficacy was evaluated comprehensively in vitro and in vivo. We observed that ADM/oHA-ss-PTX exhibited a more significant inhibitory activity on the proliferation of A549 cells compared to MCF-7 cells. This may be related to the rate of cell proliferation due to cell resistance [42,43]. High proliferation of A549 cells makes them more prone to mitotic inhibition by PTX. Moreover, the synergistic effect of ADM/oHA-ss-PTX micelles was more prominent than ADM+PTX. According to the competitive antagonism experiment, A549 cells saturated with oHA had lower ADM uptake than A549 cells without oHA treatment. It is demonstrated that ADM/oHA-ss-PTX micelles can uptake by CD44-mediated endocytosis.

In this research, particular emphasis has been placed on scrutinizing the toxicities associated with PTX and ADM. To investigate the cell biocompatibility of ADM/oHA-ss-PTX, the proliferation inhibition of normal cells (ECs) was evaluated by a CCK-8 assay. Although ADM/oHA-ss-PTX micelles at high concentrations have a killing effect on ECs, when compared with ADM+PTX, the toxicity of ADM/oHA-ss-PTX micelles is significantly milder. This indicated that the ADM/oHA-ss-PTX micelles can effectively reduce the toxicity of the PTX and ADM. In vivo experiment, throughout the treatment period, no mortality was observed in the group treated with ADM/oHA-ss-PTX micelles. However, two mice died in the ADM+PTX group. It demonstrated that the ADM/oHA-ss-PTX micelles possess the capacity to mitigate the toxicity. We did not observe insignificant body weight losses treated with ADM/oHA-ss-PTX micelles, indicating that micelles were well tolerated at the tested dosage level. Based on these results, we think this study addressed the tradeoff between PTX and ADM co-delivery efficiency and toxicities of the system.

## 5. Conclusions

In conclusion, the amphiphilic prodrug oHA-ss-PTX was successfully synthesized and characterized using ^1^H-NMR and FT-IR spectra. Subsequently, an ADM-loaded oHA-ss-PTX polymeric micelle system, which serves as a co-delivery system for ADM and PTX, was meticulously designed and successfully prepared in one step by a microfluidic process. The co-loaded micelles possessed a dynamic size (127 ± 1.4 nm, zeta potential −9.0 mV), and a high drug loading content of approximately 21.2% (PTX) and 7.6% (ADM). In vitro drug release studies showed that the release rates of both ADM and PTX were increased under high-GSH conditions. Hemolysis activity analysis, BSA adsorption testing, and cell viability assay in endothelial cells showed that the ADM/oHA-ss-PTX micelles can effectively reduce the toxicity of the PTX and ADM. Furthermore, co-loaded micelles can inhibit the activity and migration of cancer cells, have reduction-responsive properties and active targeting ability, as well as greater antitumor efficacy in vivo. This study thus addressed the tradeoff between PTX and ADM co-delivery efficiency and toxicities of the system. Overall, this study suggested that PTX-loaded oHA-ss-PTX micelles have promising potential for combination treatment of cancer.

## Data Availability

The data generated during the current study are available from the corresponding author on reasonable request.

## Supplementary Materials

The following supporting information can be downloaded at: https://www.mdpi.com/article/10.3390/pharmaceutics17040538/s1, Figure S1. FT-IR spectra of oHA-ss-NH_2_; Figure S2. ^1^H-NMR of oHA-ss-NH_2_; Figure S3. FT-IR spectra of 2′sPTX; Figure S4. ^1^H-NMR of 2′sPTX; Table S1. Effects of different PTX loading rates on the physicochemical properties of micelles; Table S2. Loading contents of ADM in ADM/oHA-ss-PTX micelles at different PTX loading; Table S3. Particle size of ADM/oHA-ss-PTX micelles under different microfluidic flow rates.
