# Supplementary material for: Development of GSH-Stimuli-Responsive Micelles Using a Targeted Paclitaxel Prodrug for Enhanced Anticancer Effect
Source: Pharmaceutics. 2025 Apr 21;17(4):538. doi: 10.3390/pharmaceutics17040538 (PMC12030733; doi:10.3390/pharmaceutics17040538)
Supplement: Supplementary file 1 [file pharmaceutics-17-00538-s001.zip › pharmaceutics-3560284-supplementary.pdf]

Development of GSH-Stimuli-Responsive Micelles Using a Targeted Paclitaxel Prodrug for Enhanced Anticancer Effect

Qian Ning [1,2], Guangping Yu [2,3], Wenkai Yi [2], Minhui Gu [2], Qianqian Xu [2] , Zhiting Ye [2], Mengxia Zhang [2,4,*], Shengsong Tang[1,2,3,5, *]

1. College of Bioscience and Biotechnology, Hunan Agricultural University, Changsha 410127, China
2. Hunan Province Key Laboratory for Antibody-Based Drug and Intelligent Delivery System, School of Pharmaceutical Sciences, Hunan University of Medicine, Huaihua 418000, China
3. Institute of Pharmacy & Pharmacology, School of Pharmaceutical Science, University of South China, Hengyang 421001, China
4. Department of Histology and Embryology, Hunan University of Chinese Medicine, Changsha 410128, China
5. Department of Pharmacology, Ningxia Medical University, Yinchuan 750004, China

* Correspondence: tangshengsong@hunau.edu.cn (S.T.); 004387@hnucm.edu.cn (M.Z.)

Supplementary Results

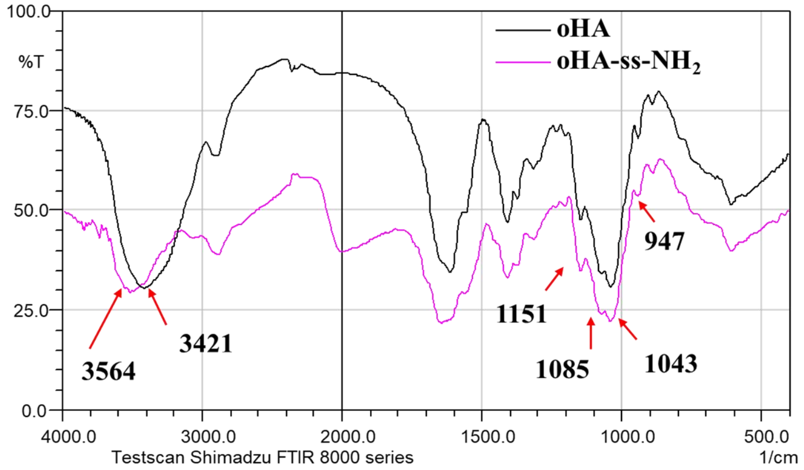

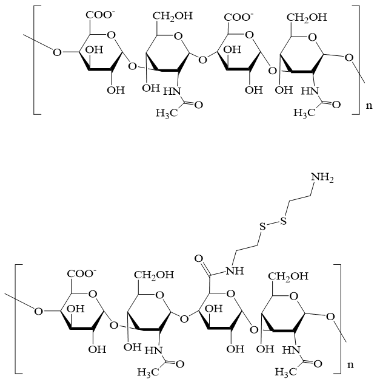

Figure S1. *FT-IR spectra of oHA-ss-NH$_2$.*

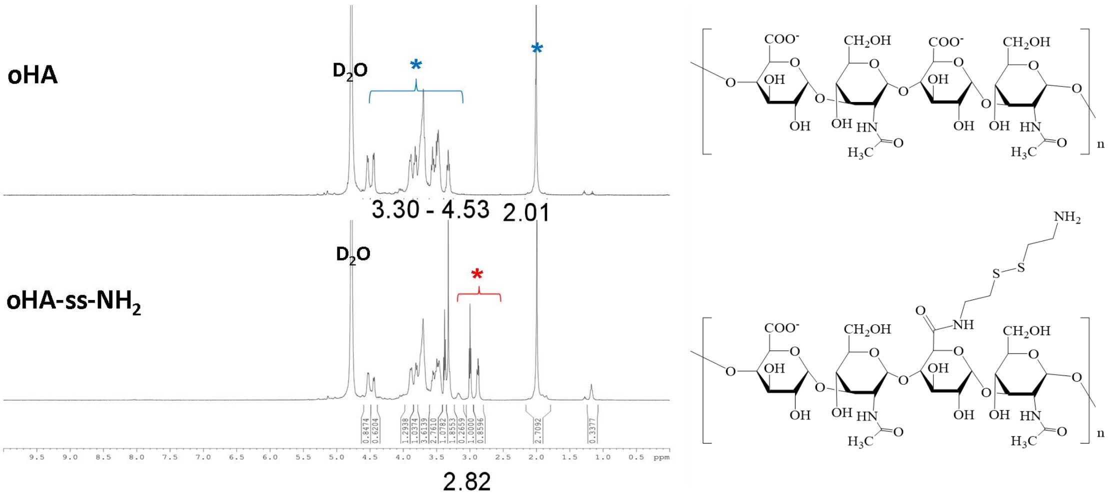

Figure S2. *^{1}H-NMR of oHA-ss-NH_2.*

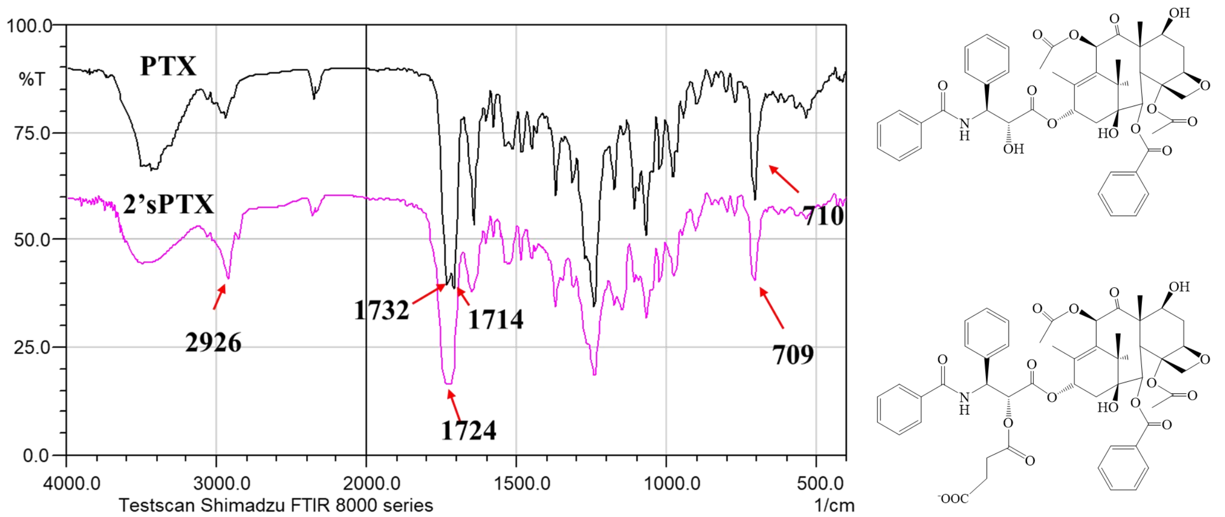

Figure S3. *FT-IR spectra of 2′sPTX.*

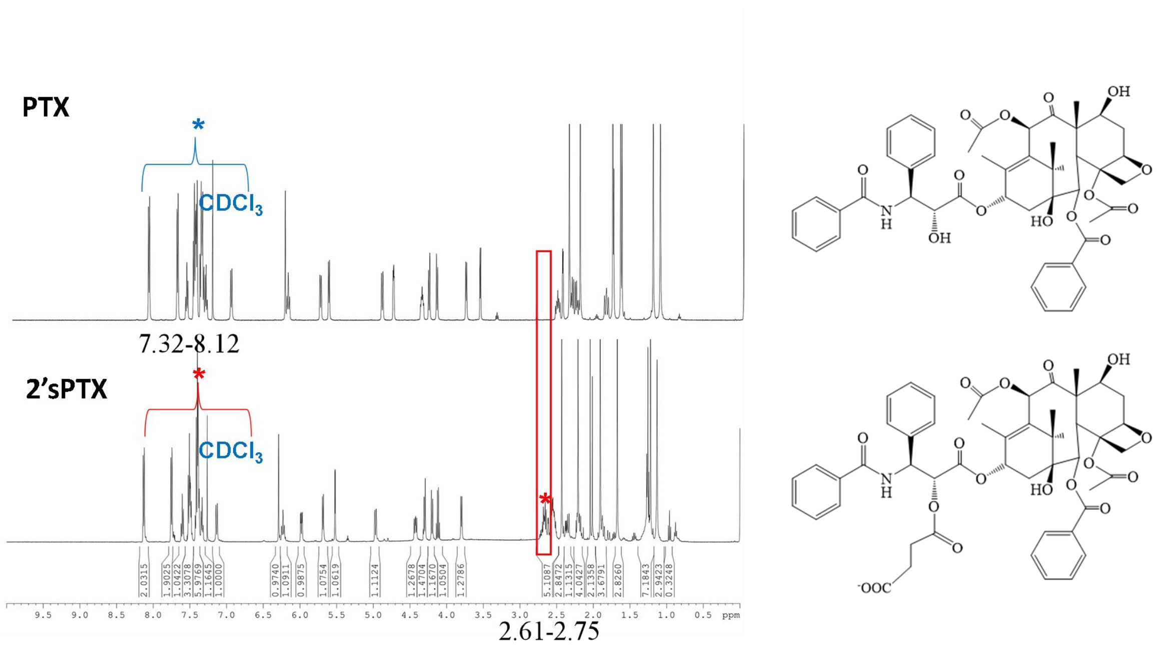

Figure S4. *^{1}H-NMR of 2´sPTX.*

Table S1. *Effects of different PTX loading rates on the physicochemical properties of micelles (n=3)*

**Mass ratio (2´sPTX/oHA-ss-NH_2)**	**LC% of PTX**	**Particle size (nm)**	**PDI**	**Zeta potential (mV)**
16:20	22.8±1.63	107±2.4	0.226±0.011	- 20.9±1.5
13:20	14.0±0.83	127±1.6	0.215±0.014	- 18.3±2.1
10:20	10.2 ±0.96	126±1.3	0.233±0.017	- 22.5±1.0
7:20	4.8 ± 0.18	174±1.2	0.278±0.013	- 19.4±3.1

Table S2. *Loading contents of ADM in ADM/oHA-ss-PTX micelles at different PTX loading (n=3)*

LC% of PTX	EE% of ADM	LC% of ADM	Particle size (nm)	PDI	Zeta potential (mV)
21.2 ± 2.3	82.3 ± 4.7	7.6 ± 0.2	127± 1.4	0.13 ± 0.038	-9.0 ± 2.0
14.0 ± 0.9	88.7 ± 2.1	5.8 ± 0.5	173 ± 2.8	0.11 ± 0.044	-13.3 ± 1.9
9.0 ± 0.6	68.3 ± 3.1	3.3 ± 0.7	189 ± 3.7	0.162 ± 0.022	-13.0 ± 1.0
5.2 ± 0.3	71.1 ± 7.7	2.3 ± 0.6	218 ± 4.9	0.20 ± 0.023	-11.8 ± 1.2

Table S3. *Particle size of ADM/oHA-ss-PTX micelles under different microfluidic flow rate (n=3)*

Flow rate (mL/min)	volumeaqueous phase/organic phase	Particle size (nm)	PDI
4	3:1	127± 1.4	0.130 ± 0.038
6	3:1	163 ± 3.9	0.210 ± 0.004
8	3:1	180 ± 6.9	0.162 ± 0.022
10	3:1	207 ± 4.8	0.30 ± 0.023
12	3:1	248 ± 17.5	0.40 ±0.035
